# High Resilience and Fast Acclimation Processes Allow the Antarctic Moss *Bryum argenteum* to Increase Its Carbon Gain in Warmer Growing Conditions

**DOI:** 10.3390/biology11121773

**Published:** 2022-12-06

**Authors:** Emma L. Gemal, T. G. Allan Green, S. Craig Cary, Claudia Colesie

**Affiliations:** 1 Global Change Research Institute, School of GeoSciences, University of Edinburgh, Edinburgh EH9 3FE, UK; 2 Department of Physical Geography, Stockholm University, SE-106 91 Stockholm, Sweden; 3 International Centre for Terrestrial Antarctic Research, University of Waikato, Hamilton 3240, New Zealand; 4 Unidad de Botánica, Facultad de Farmacia, Universidad Complutense, E-28040 Madrid, Spain

**Keywords:** Antarctica, climate change, mosses, non-vascular vegetation, acclimation, carbon gain, closed-top chamber experiment, Cape Hallett, resilience

## Abstract

**Simple Summary:**

Temperatures are increasing globally, but polar regions (including Antarctica) are warming much faster than the rest of the globe. Increased temperatures in Antarctica can impact the distribution and performance of plants, the majority of which on this continent are mosses. This study aims to investigate whether *Bryum argenteum* var. *muticum*, a moss species found in Antarctica, is capable of acclimation (adjustment of its physiology, specifically photosynthesis and respiration) to increased temperatures. We used short-term warming experiments that mimicked heatwaves and compared them to seasonal rates of photosynthesis and respiration in order to better understand how resilient this important moss species is to climate change. We found that this moss can acclimate very quickly (within 7 days) by increasing its photosynthesis (carbon gain). This shows that *B. argenteum* is highly resilient, and it may potentially benefit from short- and long-term climatic changes.

**Abstract:**

Climate warming in Antarctica involves major shifts in plant distribution and productivity. This study aims to unravel the plasticity and acclimation potential of *Bryum argenteum* var. *muticum*, a cosmopolitan moss species found in Antarctica. By comparing short-term, closed-top chamber warming experiments which mimic heatwaves, with in situ seasonal physiological rates from Cape Hallett, Northern Victoria Land, we provide insights into the general inherent resilience of this important Antarctic moss and into its adaptability to longer-term threats and stressors associated with climate change. Our findings show that *B. argenteum* can thermally acclimate to mitigate the effects of increased temperature under both seasonal changes and short-term pulse warming events. Following pulse warming, this species dramatically increased its carbon uptake, measured as net photosynthesis, while reductions in carbon losses, measured as dark respiration, were not observed. Rapid growth of new shoots may have confounded the effects on respiration. These results demonstrate the high physiological plasticity of this species, with acclimation occurring within only 7 days. We show that this Antarctic moss species appears to have a high level of resilience and that fast acclimation processes allow it to potentially benefit from both short-term and long-term climatic changes.

## 1. Introduction

Scientific evidence points clearly and convincingly to the fact that, due to anthropogenic emissions of CO_2_, Antarctica is experiencing significant environmental change [1]. Climate warming poses major challenges for plant survival, productivity and resilience on a global scale [2,3,4,5,6]. A key hypothesis in this context is that long-term adaptation to thermal conditions over multiple generations is likely to determine the degree of thermal resilience to warming at a population-specific level [7]. Presuming that selection has taken place within different thermal regimes over generations, the naturally endured temperature ranges can be explored as an estimate of thermal resilience, or sensitivity, to changing conditions: i.e., phenotypic plasticity rather than long-term adaptation. Populations that are naturally exposed to a wide temperature range have greater resilience to acute temperature changes than those deriving from climatically stable environments, a theory known as the climate variability hypothesis [8].

The terrestrial environments in continental Antarctica experience some of the starkest seasonal changes on the planet. Changes in light conditions range from three months in total darkness during winter to fully exposed to high light environments over the austral summer [9]. Vegetation is covered by snow for up to eight months during the winter, while the radiation input during the summer can heat ground temperatures up to e.g., 35.2 °C when the vegetation is active, as seen at sites such as Granite Harbour in continental Antarctica [9]. During the Antarctic winter, temperatures can fall to extreme lows, with the lowest temperature ever recorded at the surface of the Earth recorded in Antarctica (−89.2 °C; [10]). Given this naturally occurring, wide range of environmental conditions (and following the hypothesis above), we would expect that the vegetation present in these environments should have a high thermal resilience.

Resilience is understood to be the amount of disturbance an ecosystem can withstand without changing processes and structure. On the organismic level, the process that offers a mechanism for resilience is physiological plasticity. Generally, high physiological plasticity increases resilience, and this has been shown for various organisms in the past (lizards [11], Seagrass [12], bivalves [7], corals [13] and fish [14]). In plants, physiological plasticity incorporates changes of key processes such as net photosynthesis (NP) and dark respiration (DR), both of which are temperature sensitive. Traditionally, it was thought that both processes would rise exponentially with short-term changes in temperature [15]. In recent years, these models were re-evaluated because many plants show physiological, structural and biochemical adjustments that mitigate the effects of temperature increases [16,17]. This effect is referred to as thermal acclimation [18,19,20]. In the absence of thermal acclimation, metabolic reactions double in rate with an increase of 10 °C (Q_10_ rule; [21]), but if acclimation occurs, these effects are mitigated and the rates at identical test temperatures of plants grown at higher temperatures are the same as those grown at the lower temperatures [22,23].

The conventional way to test for acclimation is to monitor changes in physiological rates across the growing season [24] or to grow plants under fully controlled laboratory conditions to simulate change [8,25,26]. Measurements across a full growing season are time consuming and, especially in Polar Regions, not often feasible because of significant logistical effort. Fully controlled growth chamber experiments, on the other hand, are limited because they cannot reflect the natural variability of in situ conditions, and non-vascular vegetation, in particular, is difficult to maintain in growth cabinets [27]. Greenhouse or chamber experiments can be used to bridge that gap, especially in Antarctic environments [28]. Commonly, open-top chambers and passive warming are used [29,30,31]; however, such methods are limited by their lack of microclimate control [32]. Instead, manipulation of experimental conditions inside the chamber via heating devices allows for semi-controlled simulation of climate change, while also allowing the plants to remain in situ and exposed to natural light conditions [33,34]. We theorised that such semi-controlled chamber experiments could be used to simulate a short-term, ‘pulse’ warming event, allowing an efficient measurement of physiological plasticity and acclimation rate in Antarctic mosses.

In Antarctica’s terrestrial ecosystems, mosses play a key role and are a major component of the vegetation. Presently, a total of 109 taxa of moss (107 species + 2 varieties) are described to occur in the Antarctic Peninsula region, and 24 taxa (23 species + 1 variety) are described to occur in the continental Antarctic region [35]. Antarctic mosses have well-developed stress tolerance features, especially with regard to adjusting their photosynthetic electron transport rate under extreme environmental conditions [36]. Optimal temperatures for net photosynthesis can be as high as 15.9 °C, and gross photosynthesis is not declining even at 30 °C [9,37], yet they grow and survive even at temperatures around 0 °C [9,38,39]. Mosses have evolved mechanisms to withstand exposure to high light and UV [40,41,42,43,44,45], and, because all Antarctic species are poikilohydric, they can resist repeated and long-term desiccation [38,46,47]. Cryptobiosis (a metabolic state of desiccation and freezing induced dormancy, which can be reversed when environmental conditions become hospitable again), can allow for millennial-scale survival and viability [48,49].

Unravelling the plasticity and acclimation potential of mosses in Antarctica is necessary for a better understanding of their resilience to current climatic changes. This study aims to describe acclimation processes of DR and NP to changing temperatures with special regard to differences in their thermal acclimation potential and rate. We chose a semi-controlled in situ chamber experimental setup to test the following three statements:

1. Acclimation processes in Antarctic mosses are fast: short-term, ‘pulse’ chamber experiments can be used to simulate seasonal changes.
2. Acclimation processes in Antarctic mosses mitigate negative effects of increased temperatures through thermal acclimation of DR.
3. Physiological plasticity and acclimation potential will provide insights into the general inherent resilience of Antarctic mosses and into their adaptability to longer-term threats and stressors.

## 2. Materials and Methods

### 2.1. Study Site

**Figure 1:**
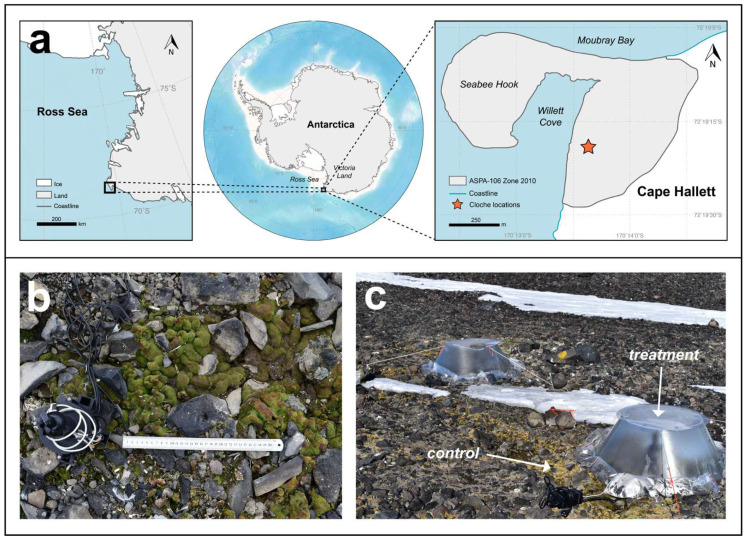
Study site location and chamber setup. (**a**) Cape Hallett location on the Ross Sea coastline in northern Victoria Land, Antarctica. (**b**) *Bryum argenteum* var. *muticum* occurs in large patches (30 cm ruler as reference). A heating device equipped with a temperature sensor and ground temperature probe (on the left) was used to control the climate in the chambers used for the warming experiment. (**c**) Two chambers were set up and secured to the ground. Mosses inside the chambers represent the treatment samples and mosses outside the chambers were the control samples.

The study was carried out at Cape Hallett (72°19′19” S, 170°13′38” E), located on the Ross Sea coastline at the southern end of Moubray Bay in northern Victoria Land, Antarctica (Figure 1a). The area around Cape Hallett is protected as Antarctic Specially Protected Area (ASPA) No. 106 (https://www.nsf.gov/geo/opp/antarct/aca/nsf01151/aca2_spa106.pdf, accessed on 1 November 2022). The area contains a low-lying spit composed of basaltic sand, gravel, and rock deposited by coastal currents, and a scree slope with a northwest aspect that rises to roughly 300 m above sea level where an extensive snow field exists. In front of the scree slope is an almost flat outwash area fed by meltwater from the snow fields above the scree. A fuller description is available in Green et al. [50] and Hofstee et al. [51].

Cape Hallett is one of the more biodiverse sites in the Ross Sea region, containing 46 lichen species, 9 bryophyte species [50], a rich arthropod community [52], a large, over 60,000 pairs, Adélie penguin (*Pygoscelis adeliae*) colony and nesting Antarctic skuas (*Stercorarius maccormicki*) [53]. Approximately half of the ice-free area at Cape Hallett is occupied by the Adélie penguin colony and is consequently devoid of vegetation [50].

### 2.2. Climate

Maximum air temperatures are above 0 °C almost every day from mid-December to the end of January [51]. The mean summer temperature of the period from 1965 to 2018 was −4.83 ± 1.68 °C, with a minimum temperature of −27.22 °C and a maximum temperature of 5.97 °C [54]. Snowfall totals about 1755 mm (actual snow depth) and falls on 160 days each year (averages for the years 1957 to 1963; [55]). Most snow falls in late summer (February–April) and least in the early summer (September–December). The northwest, a relatively protected aspect of the locality, means that soil temperatures are warm and, on Seabee Hook at 32 cm depth, are above freezing in January [51]. The depth to ice cement is greater at Cape Hallett (~80 cm) than at any other location in the McMurdo Sound region, where it is normally no more than 60 cm deep [51,56]. Surface water is present for most of December and January. Climate data for Cape Hallett are available on the LGP (Latitudinal Gradient Project) website (www.lgp.aq, accessed on 15 July 2022), and a detailed summary is provided by Green et al. [50].

### 2.3. Investigated Species

The species used in this study was *Bryum argenteum* Hedw. var. *muticum* Brid (Figure 1b), a cosmopolitan moss, although the var. *muticum* variety is restricted to polar regions and montane elevations in northerly latitudes [57]. In Antarctica, this variety is widespread across Victoria Land [58] and is the dominant moss species found in Cape Hallett [59]. Its broad distribution across Antarctica has resulted in it being a widely studied species. *B. argenteum* has historically been a source of taxonomic confusion especially within Antarctica, where *Bryum argenteum* var. *muticum* has previously been referred to as *Bryum subrotundifolium* (e.g., [9,60]). However, molecular studies have shown no difference from *B. argenteum* [61], so that the use of *B. subrotundifulium* is no longer valid [35].

### 2.4. Pulse Warming Experiment

From 25 November 2018 to 1 December 2018, an in situ pulse warming experiment was performed at Cape Hallett. Two 1 m wide fiberglass chambers were placed over patches of *Bryum argenteum* var. *muticum* growing along a running meltwater stream (Figure 1c). Each chamber was equipped with a custom-built device used to monitor the microclimate (air temperature, soil temperature and relative humidity) inside and outside and to warm up and control the temperature inside the chambers. The electric fan at the bottom of the instrument circulated air across a heating element, controlled via an internal feedback-loop, which was programmed to keep internal chamber temperatures at 5 °C warmer than ambient air temperatures, and to keep chamber temperatures ≥0 °C overnight. Spot measurements using a portable chlorophyll fluorometer (Junior-PAM, Heinz Walz GmbH, Effeltrich, Germany) showed the mosses to be continuously active over the test period, as has also been reported for another moss species, *Hennediella heimii*, in midsummer at Canada Glacier flush, Taylor Valley [62]. At the conclusion of the experiment, three patches of moss were collected from inside each chamber (treatment samples), and three patches of moss were collected from outside and adjacent to each chamber (control samples). Sample size: 3 treatment × 2 chambers + 3 control × 2 chambers = 12 moss samples. All samples were air dried, frozen and transported to the laboratory, where they were stored frozen (−20 °C) until used. Frozen storage is considered a suitable long-term storage method of mosses [63].

Spot measurements of incident photosynthetic active radiation inside and outside the chamber were performed with a handheld light meter (LTD SKP200, Skye Instruments, Llandrindod Wells, UK) and compared using a paired *t*-test. A sample of the chamber material was analysed for light transmission at the NERC Field Spectroscopy Facility’s optical laboratory. A Hoffmann Engineering LS-65-GF luminance standard with a 350–1100 nm spectral range was used as an illumination source. Radiance was recorded using an Ocean Insight FLAME UV-Vis (200–850 nm) spectrometer, with and without the chamber material present between the standard and spectrometer. Transmittance was calculated as the light received at the spectrometer directly from the lamp divided by the light received after passing through the chamber material.

#### 2.4.1. Gas-Exchange Measurements

CO_2_ gas exchange measurements were conducted using a minicuvette system (CMS400, Walz GmbH, Effeltrich, Germany). The response of net photosynthesis (NP) and dark respiration (DR) was measured under optimal water contents. To determine the optimal water contents, complete desiccation cycles were performed (saturated to air dry) for each sample and during which the samples were weighed at intervals and water content (WC) determined as percentage of the dry weight. Ninety percent of maximum NP (NP_max_) was identified as the lower threshold for optimal sample hydration. Once the optimal water content was known, samples were exposed to varying conditions of light intensity and temperature, described in further detail in the experimental procedures below. Rates of NP and DR were calculated from the CO_2_ exchange measurements using the equation described by Farquhar and von Caemmerer [64]. All calculations for NP and DR rates derive from those measurements were based on a sample canopy surface area.

#### 2.4.2. Pulse Warming Event Measurements

All samples, treatment and control from the pulse warming experiment underwent a reactivation procedure prior to the gas-exchange measurements in the laboratory. For each set of measurements, three samples at a time were taken out of the freezer and stored in a fridge at 8 °C for 24 h. Once thawed, the samples were carefully rewetted and kept in the fridge at low-light (25 µmol photons m^−2^ s^−1^) for a further three days. Samples were also stored in the fridge between gas-exchange measurements.

To measure the gas-exchange responses to incident light, samples at optimal water content (determined in preliminary drying experiments) were exposed to increasing light from 0 to 1500 µmol photons m^−2^ s^−1^ at a constant temperature of 15 °C (optimum temperature previously reported for *B. argenteum* in Cape Hallett; [9]) and ambient CO_2_ concentration until NP saturation was reached at each new light interval. Photosynthetic photon flux density (PPFD) response curves were obtained using non-rectangular hyperbola models [65], which provided the light compensation point (LCP; the lowest light intensity at which positive NP is reached) at the point intersection between the calculated curve and the *x*-axis. Light saturation points (LSP; defined here as the light intensity at which 90% of NP_max_ is reached) were also calculated from the response curves.

To measure the gas-exchange responses to different temperatures, samples at optimal water content were illuminated at their respective LSPs and their gas-exchange measured across a series of temperature steps (2, 5, 10, 15, 20, 25 and 30 °C). DR was measured directly before each reading in the light (the NP reading) by darkening the cuvette with a plastic lid. The order of temperature steps was randomized to avoid potential acclimation to gradually changing temperatures. From these measurements, gross photosynthesis (GP) was calculated as the sum of NP and DR. Mean (± standard error) NP, DR and GP rates were then plotted for each temperature step.

### 2.5. Seasonal Measurements

During a visit to Cape Hallett from November 2004 to January 2005, CO_2_-exchange of *B. argenteum* var. *muticum* was measured 5 times across the summer season (17 November 2004, 2 December 2004, 20 December 2004, 2 January 2005 and 19 January 2005) using a minicuvette system (CMS400, Walz GmbH, Effeltrich, Germany). Five replicates of moss growing on the outwash area were selected and, on the sampling dates, cores (5 cm diameter, 1 cm thick) were collected for the measurements. The samples were wetted and stored for 12 h in light at >0 °C. For the gas-exchange measurements, the temperature in the cuvette was set to 10 °C and rates of DR (dark) and NP (25, 50, 100, 200, 400, 800, 1000, 1250 and 1500 µmol photons m^−2^ s^−1^) were measured. From these measurements, GP (NP + DR) was calculated and the mean (± standard error) NP_max_, DR and GP across the season were then plotted against time.

Hourly air temperature data for the measurement period were available from the long-term climate monitoring station at Cape Hallett (https://amrc.ssec.wisc.edu/, accessed on 4 September 2022).

### 2.6. Measurements of Chlorophyll Contents

Chlorophyll content of each sample (for both the pulse warming measurements and seasonal measurements) was determined at the completion of the CO_2_-exchange measurements by extracting the samples twice with dimethyl-sulfoxide (DMSO) at 60 °C for 90 min and then measuring the absorption at standard wavelengths [66].

### 2.7. Calculations and Statistical Analysis

Calculations and statistics were performed using RStudio (version 4.0.2; R Core Team, 2021). All the figures were produced using the ‘ggplot2’ package [67]. The data and code to reproduce the data analysis and plots can be found on GitHub at: https://github.com/emmagemal/BryumAcclimation (accessed on 4 November 2022).

#### 2.7.1. Cardinal Points of Photosynthesis and Respiration

From the pulse warming experiment, control and treatment values of cardinal points for photosynthesis (NP_max_, optimum temperatures, upper threshold temperatures for negative NP, light saturation and light compensation points) were extracted from the temperature and light response curves respectively using the ‘retistruct’ package in R [68]. Optimum temperature was defined as the temperature at mean NP_max_. The upper threshold temperature for negative NP was defined as the temperature at NP = 0 µmol m^−2^ s^−1^. The upper threshold temperature for negative NP and the light saturation and compensation points were compared between control and treatment groups using Student’s *t*-tests.

Rates of NP and DR from those samples as a function of temperature were compared between control and treatment groups. After testing for normality and equal variances, an analysis of covariance (ANCOVA) was conducted using mixed effects models (created with the ‘lme4’ package; [69]), with NP and DR considered separately due to their different relationships with temperature. Temperature was the continuous covariate and variation between samples was controlled for. The data used passed normality tests (Shapiro-Wilk). Models were compared using Akaike Information Criterion (AIC) values [70], with the model with the lowest AIC value considered the most parsimonious. If two models differed by <2 AIC values, then the models were considered equivalent. The ANCOVA was performed with the most parsimonious model, using Type III error and a significance level of *p* < 0.05.

#### 2.7.2. Carbon Use Efficiency

Carbon use efficiency (CUE) is broadly defined as the proportion of carbon acquired from the environment that is used for growth [71,72,73]. Here, an instantaneous measure of carbon allocation is used and is defined as CUE = NP/GP = NP/(NP + DR), typically given as a percentage, and was calculated for control and treatment samples at each temperature step. High CUE favours photosynthesis, whereas low CUE favours respiration. CUE at each temperature step were compared between control and treatment samples using *t*-tests.

#### 2.7.3. Acclimation Potential

The acclimation potential of NP and DR, the acclimation ratio (AR), was calculated by dividing rates from the treatment samples at growth temperature (5 °C higher than controls) by rates from the control samples at growth temperature (Rate_treatment at T+5°C_/Rate_control at T_) at each temperature step. According to Larigauderie and Koerner [22], an AR of 1.0 indicates acclimation, i.e., the DR or NP at the identical test temperature of plants grown at the higher temperature is the same as those grown at the lower temperature. For DR, values greater than 1.0 indicate partial or no acclimation. In situations where acclimation is occurring, the DR temperature response curve of the treatment samples would be lower than the control rates at any selected temperature. If partial acclimation has occurred, then AR will be less than control group Q_10_ for the same temperature range [22]. Q_10_ values for the DR response curves were calculated using an interactive table at https://www.physiologyweb.com/calculators/q10_calculator.html (accessed on 3 August 2022). For NP, acclimation is indicated by identical or greater rates of the treatment samples in comparison to the controls, with potential shifts in the temperature response curve [74]. Therefore, values greater than 1.0 indicate acclimation through increased rates (potentially in combination with shifted response curves) while values less than 1.0 indicate a lack of acclimation.

## 3. Results

### 3.1. Pulse Warming Experiment

#### 3.1.1. Conditions

**Figure 2:**
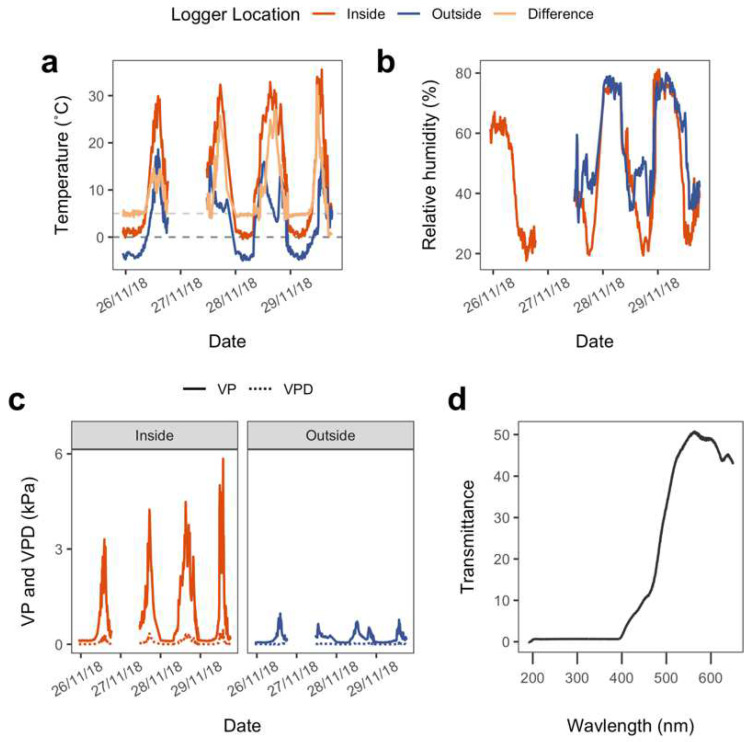
Conditions during the pulse warming experiment, for four days during the period 26th to 29th of November 2018. (**a**) Temperature inside the chamber (blue line), temperature outside the chamber (red line) and the difference between inside and outside temperatures (light orange line) in °C; (**b**) comparison of relative humidity in % inside (blue line) and outside (red line) the chamber; (**c**) water vapour pressure (VP, solid line) and water vapour pressure deficit (VPD, dotted line) in kPa for inside (treatment, blue lines) and outside (control, red lines) the chamber; (**d**) UV-VIS transmission spectra of the chamber material.

During the experimental manipulation (warming treatment), the treatment group was covered by a closed-top chamber while the control group was exposed to ambient conditions (Figure 1c). The chamber had an automatic heating system designed to keep internal air temperatures 5 °C warmer than the outside air. This system was successful overnight (2100 to 0800, approx.) and the temperature difference between inside and outside temperatures was 4.88 ± 0.11 °C. During the day, the chamber showed extensive warming due to incident radiation and internal air temperatures could reach 25 °C warmer than the external air (absolute values around 32 °C), indicating that the warming effect of the chamber was much larger than the planned 5 °C (Figure 2a).

Data for relative humidity (RH) were only available for 3 days but showed a similar pattern to temperature (Figure 2b). During the night, there was no significant difference in RH between inside and outside the chamber (63.2 ± 1.2% inside vs. 64.1 ± 1.1% outside). During the day, and most likely driven by the temperature difference, RH was significantly higher outside the chamber than inside (31.0 ± 0.8% inside vs. 45.7 ± 0.7% outside; *t* = 13.98, df = 270, *p* < 0.0001). The water vapour content of the air was about 50% higher inside the chamber overall (means 0.65 ± 0.01 kPa inside, and 0.43 ± 0.01 kPa outside) (Figure 2c). Additionally, most likely due to the higher temperatures, the mean vapour pressure deficit inside was 260% higher during the day (1.12 ± 0.05 inside the chamber, compared to 0.42 ± 0.02 kPa outside). Absolute maxima were also much higher inside compared to outside (4.45 kPa inside vs. 1.23 kPa outside).

The photosynthetic active radiation was significantly reduced inside the chamber (941 ± 65 inside vs. 1051 ± 101 outside; *t* = 3.64, df = 11, *p* = 0.003). Spectrophotometric scans of the chamber material showed it to be almost completely opaque at wavelengths below 400 nm and effectively shaded against UV-radiation (Figure 2d).

#### 3.1.2. Physiological Responses

**Figure 3:**
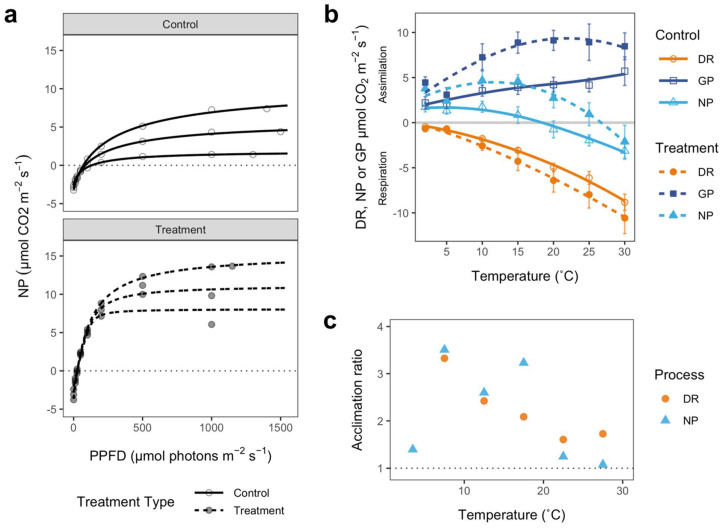
Physiological responses of *B. argenteum* var. *muticum* in response to the pulse warming experiment. (**a**) Light response curves for control (top) and treatment (bottom); (**b**) the response of net photosynthesis (NP, light blue ▲), dark respiration (DR, orange ●) and gross photosynthesis (GP, dark blue ■) to temperature. The symbols are actual data means ± SE at each temperature, with hollow symbols for control values and filled symbols for treatment values. The fitted lines (solid for control and dashed for treatment) were obtained from nonlinear curve fitting of the entire data set in R. NP: control, R^2^ = 95.8%, *p* = 0.0008; treatment, R^2^ = 87.6%, *p* = 0.0068. DR: both control and treatment, R^2^ = 99.2%, *p* < 0.0001; (**c**) acclimation ratios for NP (light blue ▲) and DR (orange ●) at various temperatures, calculated from average treatment and control rates per surface area. Dotted line indicates an acclimation ratio of 1.0 (acclimation as identical rates of control and treatment at respective growth temperatures).

The treatment group of mosses differed significantly from the uncovered controls in several CO_2_-exchange parameters related to incident light. The treatment group had lower light compensation points (28.00 ± 2.89 vs. 94.67 ± 5.21 µmol m^−2^ s^−1^ for treatment and control respectively; *t* = 11.20, df = 4, *p* = 0.0004), lower light requirement to achieve maximal NP (LSP; 256.7 ± 14.5 vs. 518.8 ± 54.5; *t* = 4.48, df = 4, *p* = 0.011), a higher quantum efficiency for CO_2_ uptake (0.11 ± 0.003 vs. 0.035 ± 0.009; *t* = 20.79, df = 4, *p* < 0.0001), and higher mean maximal rates of net CO_2_ exchange (10.57 ± 1.49 vs. 3.23 ± 1.50; *t* = 3.23, df = 4, *p* = 0.032) (Figure 3a). Two of the three treatment replicates showed a decline in NP at light intensities above 1000 µmol m^−2^ s^−1^, a feature not seen in any of the controls. The treatment group also had significantly higher chlorophyll contents than the control group (1300 ± 190 vs. 740 ± 120 mg m^−2^; *t* = 2.49, df = 8, *p* = 0.037).

The response of NP to temperature followed the same pattern for treatment and control samples. The rates of NP first increased to an optimum temperature, beyond which the rates declined until they became negative (Figure 3b, light blue triangles). The mean threshold temperature for negative NP was significantly higher in the treatment group (26.9 ± 1.8 °C vs. 17.9 ± 2.3 for treatment and control respectively; *t* = −3.02, df = 9, *p* = 0.014). Additionally, the maximal rate of NP at optimal temperature was greater for the treatment group (4.38 vs. 1.71 µmol m^−2^ s^−1^) and the optimal temperature for NP was much higher in the treatment group compared to the controls (11.65 °C vs. 3.03 °C). Rates of DR increased exponentially with temperature for both groups (Figure 3b, orange circles), but the DR rates of the treatment group were consistently higher than those of the controls (28.6 ± 7.8% higher; *t* = 3.63, df = 6, *p* = 0.011). Rates of GP increased with temperature for both groups (Figure 3b, dark blue rectangles), but the GP rates of the treatment group were consistently nearly twice as high as those of the controls (94.4 ± 12.4% higher; *t* = −5.99, df = 6, *p* = 0.0097). At higher temperatures, GP of the treatment group started to decrease slightly, while control GP continued increasing.

For both groups, CUE was highest (between 78.7–85.1%) at low temperatures (2 °C) and then declined as temperatures increased (Table 1). Between 5–10 °C, CUE reached 50% for the control group, while this threshold was reached at a higher temperature for the treatment group (between 15–20 °C). A CUE of >50% indicates that NP is the dominating process in GP, while <50% indicates DR is dominating. Overall, CUE was higher in the treatment group than in the control group (average CUE of 41.2 ± 3.8% for the controls vs. 48.4 ± 4.6% for the treatments), but CUE did not significantly differ between the groups overall nor at each temperature step (Table 1).

The thermal acclimation of *B. argenteum* var. *muticum* was tested by calculating an acclimation ratio (AR) at each measurement temperature for both DR and NP (Figure 3c). A value of 1.0 indicates identical rates between control and treatment groups at growth temperature. For DR, the AR declined with increasing temperatures (from 3.33 to 1.73), in line with the increased divergence of respiration rates with temperature between the control and treatment groups (seen in Figure 3b). The Q_10_ for DR of treatment samples (10–20 °C range) was 2.49, about 11% lower than the controls. Additionally, the Q_10_ for DR of control samples (10–20 °C range) was smaller than the AR for the same temperature range (2.81 vs. 3.62), indicating no partial acclimation of treatment DR. For NP, the AR followed more of a bell curve (from 1.24 up to 3.51 and then down to 1.07). Between 5–20 °C, AR remained relatively high until after the 15–20 °C range where the ratio declined drastically thereafter. This decline is in accordance with the rapid decline in NP beyond optimal temperature (Figure 3b). At 2–5 °C and warmer temperatures beyond 15–20 °C, the acclimation ratio of NP was close to 1.0 (1.07–1.24), indicating a slight shift in the temperature response curve.

### 3.2. Seasonal Patterns

**Figure 4:**
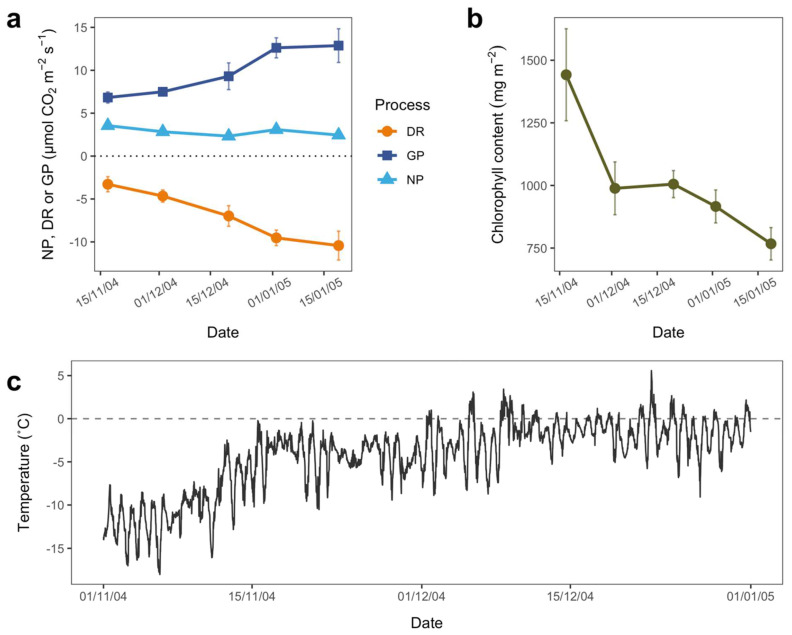
Physiological changes under ambient air temperatures in *Bryum argenteum* var. *muticum* across the Antarctic summer season November 2004 to January 2005. (**a**) changes in net photosynthesis (light blue ▲), gross photosynthesis (dark blue ■) and dark respiration (orange ●). Shown are mean values ± SE for *n* = 5 samples; (**b**) changes in chlorophyll contents (mean values ± SE for n = 5 samples); (**c**) ambient air temperature at Cape Hallett during the summer season 2004. Temperature is recorded in °C at 15 min intervals.

GP and DR of *B. argenteum* increased significantly across the summer season 2004/05 (DR Regression: 0.12, adjusted R^2^ = 0.58, F1 = 32.21, *p* = <0.0001; GP Regression: 0.11, adjusted R^2^ = 0.45, F1 = 19.49, *p* = 0.0022; Figure 4a). NP remained constant throughout, with no significant increase or decrease in rate across the season (Figure 4a; NP Regression: −0.013, adjusted R^2^ = 0.11, F1 = 3.89, *p* = 0.061). Over the same time period, the chlorophyll content in the samples declined (Figure 4b; Regression: −8.85, adjusted R^2^ = 0.36, F1 = 19.37, *p* = 0.00011).

Over the time course of this study, temperature increased significantly (Figure 4c, Regression: 2.06 × 10^−6^, adjusted R^2^ = 0.57, *p* < 0.0001), and there was large variability in temperature, with minimum temperatures reaching −18.0 °C and maximum temperatures reaching 5.6 °C. The beginning of the season (November 2004) held the majority of colder temperatures, while the middle (December 2004) to end (January 2005) of the season had on average relatively warmer temperatures.

## 4. Discussion

We found that *Bryum argenteum* var. *muticum*, a cosmopolitan moss species found in Antarctica, can acclimate to both seasonal changes and short-term pulse warming events to mitigate negative effects of increased temperature. Acclimation occurred quickly under both seasonal and pulse temperature changes. Following experimental pulse warming, net photosynthesis (NP) showed high metabolic plasticity through strong upregulation of gross photosynthesis (GP), which overcompensated for higher respiratory losses from increased dark respiration (DR). Under seasonal summer warming, rates of NP remained stable, due to a maintained balance of GP and DR rates. These results demonstrate the high physiological plasticity of this species, in particular the ability to rapidly and dramatically increase carbon uptake under warmer conditions. We show that this Antarctic moss also appears to have an inherent resilience, and can potentially benefit from both short-term and long-term climatic changes. 

Acclimation of *B. argenteum* occurred, but, in contrast to our predictions, the acclimation was not apparent for DR. Thermal acclimation of DR is a common response to long-term temperature increases in higher plants (e.g., [75]). Acclimation of DR in response to warming typically expresses itself as a decrease in rate, or occasionally respiratory homeostasis [76]; however, this did not occur here. There were some indications that DR had commenced to acclimate, but these were small. The Q_10_ for DR of treatment samples (10–20 °C range) was 2.49, about 11% lower than the controls. Additionally, the AR (acclimation ratio, DR_treatment at T+5°C_/DR_control at T_) declined from around 3.33 to 1.73 over the temperature range 2–30 °C (Figure 3c), and this decline in AR with temperature would normally indicate Type 1 acclimation [77]. However, acclimation did not actually occur because treatment DR rates were always higher than control DR (Figure 3b), and AR was greater than the Q_10_ for the 10–20 °C range [22]. In typical acclimation studies, the AR would be near 1.0 and DR of treatment samples at growth temperature always lower than DR of the controls at any chosen temperature [22]. Thus, while the potential for greater carbon losses from respiration at higher temperatures tends to drive thermal acclimation in other plants, pulse warming does not result in DR acclimation in Antarctic mosses, although there was a slight decrease in temperature sensitivity and Q_10_ of respiration.

We found that acclimation in *B. argenteum* occurred quickly, as we hypothesized, with large differences achieved between control and treatment samples after only 7 days of warming; however, these differences were in NP. The pulse temperature change led to higher overall rates of NP in the treatment group than the control NP across all the temperature steps measured (Figure 3b), as well as higher maximal rates of NP at optimal temperature (1.71 µmol m^−2^ s^−1^ for controls vs. 4.38 µmol m^−2^ s^−1^ for treatments). DR and GP were also higher for treatment samples (28.6 and 94.4%, respectively). The overall response of NP to temperature was also different, with treatment samples showing a higher mean threshold temperature for negative NP (17.9 vs. 23.8 °C, control and treatment respectively). Optimal temperatures for NP also changed markedly from 3.03 °C for the controls to 11.65 °C for the treatment samples. This was further indicated by the low AR ratios (near 1.0) at very low and high temperatures (2 °C and 25–30 °C), implying nearly identical rates between control and treatment samples (at 5 °C higher temperatures than control samples). The net result was that CUE (carbon use efficiency) fell more quickly with increasing temperature for the control group, reaching a dominance of DR in GP (CUE < 50%) between 5–10 °C, whereas, for the treatment group, this was reached between 15–20 °C (Table 1). Shifts in the optimum temperature have been described before for *B. argenteum* [9], with the thermal optimum for NP changing between summer seasons. The optimum temperatures determined for the control and treatment groups in this study fell within the lower range of the most recently reported optimums for Antarctic *B. argenteum* (9.1–15.9 °C; [9]), most likely because our pulse warming experiment was towards the beginning of the summer season before seasonal acclimation took place (late November), whilst the sampling for Pannewitz et al. [9] occurred towards the end of the season (January and February). 

These results indicate strong acclimation of NP to increased temperatures, whereby any negative consequences of temperature increases are quickly mitigated at an impressive rate (only 7 days) by increasing rates of NP and slightly shifting its thermal response upwards. Furthermore, the short but intense temperature increases applied in our pulse warming experiment (Figure 2a) appeared to benefit the mosses, with carbon uptake of the treated samples increasing by 300% (AR of 2.59 to 3.51) compared to the rates of the control group around the new temperature optimum (Figure 3c). This has important implications for the future of Antarctic moss populations and carbon feedback, particularly if the frequency of heatwaves continues to increase [78]. 

The patterns reported from the pulse warming experiment represent an almost perfect match to those reported to occur across the season. The moss showed considerable increases in both GP and DR through an Antarctic summer at Cape Hallett; however, NP remained almost stable (Figure 4a). The general improvement in growth conditions through the summer (warmer (Figure 4c), wetter) is the probable driver of these changes. Antarctic research, particularly in continental Antarctica, tends to take place over short periods, weeks rather than months. Thus, the results are a warning that such short-term measurements may not reflect the conditions across the season. Additionally, there is high site and year-to-year variation in plant performance which would compound the problem [9,79]. Researchers need take this into account, and this means that extrapolations from such studies should be made with care. However, the similarity between the pulse warming results and those across the season demonstrate that chamber experiments may be a useful tool for measuring changes when longer-term studies are not feasible.

In terms of the response of NP to light, compared to the controls, the treatment group had lower light compensation (28.0 vs. 94.7 µmol m^−2^ s^−1^ for treatment and control respectively), a lower LSP (light saturation point; 256.7 vs. 518.83 µmol m^−2^ s^−1^), a higher quantum efficiency for CO_2_ uptake (0.11 vs. 0.035) and higher mean maximal rates of net CO_2_ exchange (10.57 vs. 3.23 µmol m^−2^ s^−1^; Figure 3a). The above data strongly suggest that the treatment group became shade-acclimated. The LSP for the treatment group was on par with previous estimates of LSP for shade-acclimated *B. argenteum* (300 µmol photons m^−2^ s^−1^; [80]). What is unusual is that the treatment (potentially shade-acclimated) samples had higher maximal NP and higher DR. This is the opposite of the normal situation found in higher plant leaves where shade-acclimated leaves have both lower maximal NP and DR [81]. However, our results align with a published study on *B. argenteum* at Botany Bay, Antarctica, which shows exactly the same overall pattern for sun-adapted mosses that had been deeply shaded for 2 weeks [82]. There seems to be little doubt that this pattern reflects that of shade acclimation in *B. argenteum*.

Shade acclimation typically occurs through loss of protective pigments or the growth of new shoots, and these changes can be rapid (~5 days; [80]). The rapid growth of new shoots during the pulse warming experiment could explain the elevated activity in the treatment samples, with new tissues increasing the rates of NP and DR relative to the control group. Additionally, investment in chlorophyll due to a loss of protective pigments can also increase photosynthetic capacity. Shade-acclimated *B. argenteum* has shown dramatic increases (220%) in chlorophyll content following recovery after shading [80] and, in our study, the treatment group had double the chlorophyll content of the control group. Overall, it appears that shade acclimation did occur to some extent in the treatment group, but we cannot say the exact extent to which this masked potential thermal acclimation in DR.

Although the light levels inside the chambers were significantly reduced, the amount of the decrease was only 10.5%. This means that there was relatively little depression of light within the chambers. This depression in light quantity appears to be similar to those found in other chamber studies in Antarctica and probably elsewhere, as it is a typical published value for the materials used [83]. However, spectrophotometric scans of the chamber material show it to be almost completely opaque at wavelengths below 400 nm (Figure 2d). This was somewhat unexpected because the material has been widely used for open- and closed-top chamber experiments in Antarctica, yet UV exclusion has not been reported. Since Antarctic mosses are known to respond rapidly to changes in incident UV radiation [60,84], it is possible that UV is involved in initiating morphological changes, and this would explain the occurrence of an apparent shade-form in the chamber despite light levels being high.

The above results are interesting not only because the changes within the chamber were fast, indicating an ability for this moss to rapidly adapt to environmental change, but they may not have been driven by acclimation of existing structures, but, possibly, by the rapid formation of new shoots. Higher plants show a similar response whereby shade leaves are replaced by sun leaves when canopy opening occurs, rather than the shade leaves acclimating to the changed conditions [81]. It is important to point out that protection against high light is apparently constitutive in *B. argenteum*, so the changes are not necessarily to protect the photosynthetic pathways but are better interpreted as acclimation to improve net carbon gain despite a warming environment. All the preceding points suggest that changes in plant performance in closed- and open-top chamber experiments may not be exclusively the results of increasing temperatures, a warning which already exists in the literature [83].

With high plasticity indicated by the rapid physiological and morphological changes in *B. argenteum*, the suggestion that the extreme seasonality of Antarctica leads to high resilience is supported. Additionally, *B. argenteum* populations in Cape Hallett studied over time have remained stable [54], which further corroborates their inherent resilience to change. Although general warming trends have not yet been exhibited across continental Antarctica [85], the region is predicted to become increasingly exposed to thermal stress in the form of heatwaves [86,87]. In line with this prediction, in March 2022, large parts of East Antarctica experienced an extreme heatwave never before recorded in the more than 60 years of instrumented records [3]. In maritime Antarctica where general warming and a high frequency of heatwaves have already been experienced [86,88], moss growth rates are much higher than that in continental Antarctica, with rates of moss organic matter accumulation up to three times higher [89]. With the versatility in carbon gain seen under our pulse warming treatment, mosses in continental Antarctica are likely to increase growth rates and potentially expand in coverage or density under predicted short- or long-term warming. However, enhanced growth and expansion are dependent on how increased temperatures alter water availability and permafrost active layer depth, factors which can greatly affect moss performance [54,90].

## 5. Conclusions

Our findings provide some of the first insight into the acclimation potential and resilience of Antarctic mosses to short-term temperature increases. Processes, although not necessarily thermal acclimation, in *Bryum argenteum* var. *muticum* were fast, with effects seen within one week of experimental treatment. Acclimation of DR was not obvious and may have been confounded by the mosses creating new growth rather than acclimating existing structures. However, there are strong indications of acclimation of NP to warmer temperatures, particularly through increased rates of carbon gain. Short-term, closed-top chamber warming experiments can be used to effectively simulate seasonal changes, allowing efficient measurement of physiological plasticity and acclimation rates. Such ‘pulse’ experiments provide important insight into the general inherent resilience of Antarctic mosses, and their ability to acclimate to short- and longer-term threats and stressors. Our results for *Bryum argenteum* suggest that moss species are likely to benefit from warming events, whether in the form of heatwaves or long-term increases, through their ability to increase their carbon gain.

## Figures and Tables

**Table 1:** Carbon use efficiency (CUE) and *t*-test results of *B. argenteum* var. *muticum* in response to the pulse warming treatment. CUE is the average CUE in % ± SE across control and treatment samples respectively for each temperature step in °C measured. >50% CUE favours photosynthesis, whereas <50% CUE favours respiration.

	CUE		Significance	
Temperature (°C)	Control	Treatment	*p*	*t*
2	78.7 ± 2.1	85.1 ± 3.3	0.19	−1.65
5	64.1 ± 6.2	73.6 ± 6.8	0.33	−1.03
10	42.20 ± 9.6	60.2 ± 7.3	0.17	−1.49
15	38.0 ± 5.5	53.7 ± 6.8	0.10	−1.81
20	28.8 ± 3.14	32.6 ± 9.9	0.73	−0.36
25	21.7 ± 6.4	21.3 ± 5.7	0.97	0.38
30	25.7 ± 7.2	12.9 ± 9.3	0.35	1.08

## Data Availability

The data and code to reproduce the data analysis and plots are publicly available on GitHub at: https://github.com/emmagemal/BryumAcclimation (accessed on 4 November 2022).
